# Messing with the Sentinels—The Interaction of *Staphylococcus aureus* with Dendritic Cells

**DOI:** 10.3390/microorganisms6030087

**Published:** 2018-08-15

**Authors:** Murthy N. Darisipudi, Maria Nordengrün, Barbara M. Bröker, Vincent Péton

**Affiliations:** Department of Immunology, University Medicine Greifswald, Ferdinand-Sauerbruch-Straße DZ7, D-17475 Greifswald, Germany; venkata.darisipudi@uni-greifswald.de (M.N.D.); maria.nordengruen@uni-greifswald.de (M.N.); broeker@uni-greifswald.de (B.M.B.)

**Keywords:** *S. aureus*, dendritic cells, innate immunity, adaptive immunity, immune evasion, infection

## Abstract

*Staphylococcus aureus* (*S. aureus*) is a dangerous pathogen as well as a frequent colonizer, threatening human health worldwide. Protection against *S. aureus* infection is challenging, as the bacteria have sophisticated strategies to escape the host immune response. To maintain equilibrium with *S. aureus*, both innate and adaptive immune effector mechanisms are required. Dendritic cells (DCs) are critical players at the interface between the two arms of the immune system, indispensable for inducing specific T cell responses. In this review, we highlight the importance of DCs in mounting innate as well as adaptive immune responses against *S. aureus* with emphasis on their role in *S. aureus*-induced respiratory diseases. We also review what is known about mechanisms that *S. aureus* has adopted to evade DCs or manipulate these cells to its advantage.

## 1. Introduction

*Staphylococcus aureus* (*S. aureus*) can act as a commensal bacterium in humans, where it frequently colonizes the airways, skin and gut. In most cases, the host can maintain equilibrium with the bacteria over long time periods. However, given the opportunity, *S. aureus* can cause a broad range of infections, ranging from mild, self-limiting skin and soft tissue infections to life-threatening diseases [1]. In the airways, *S. aureus* can cause pneumonia, and colonization with the bacteria is associated with allergic airway inflammation, especially with chronic rhinosinusitis with polyps and asthma [2]. Similarly, *S. aureus* is frequently found on inflamed skin of patients with atopic dermatitis (AD) [3]. 

Although *S. aureus* mainly has an extracellular lifestyle, the microorganism is also able to invade phagocytes as well as epithelial and endothelial cells and persist intracellularly [4]. To control the bacteria and the infected cells, the host immune system uses every level of its defense mechanisms [1]. Innate and adaptive immunity are involved, and both humoral and cellular effector mechanisms are required to keep the microorganism in check. Being at the interface between innate and adaptive immune responses, dendritic cells (DCs) must be central to the immune protection against *S. aureus* [5].

Recognition of *S. aureus* by professional phagocytes, such as monocytes (MOs), macrophages (Mφs) and DCs, induces the release of cytokines and chemokines, and the latter recruit neutrophils to the site of infection. Neutrophils are essential for killing the bacteria, either by phagocytosis or by NETosis. Phagocytosis is a process of engulfing and digestion of bacteria inside the cell, whereas NETosis involves trapping of bacteria in net-like structures, called neutrophil extracellular traps (NETs), which consist of DNA and histones as well as the content of neutrophil granules, such as anti-microbial peptides (AMPs) and elastase [1]. As a counter measure, *S. aureus* secretes many proteins that interfere with both the recognition by phagocytes and their chemotaxis to the infection site [6]. The microorganism is further capable of destroying NETs by nuclease production [7].

The humoral arm of the innate immune system, the complement cascade, is also indispensable in the defense against *S. aureus*. Complement factors or their fragments can promote opsonization to facilitate microbial clearance (C3b) and drive inflammation (C3a and C5a). These functions are mediated by specific complement receptors (CRs) on host cells. Moreover, complement can kill many bacterial species directly by forming pores in their membranes, the membrane attack complexes (MACs). In return, *S. aureus* interferes with complement function at many levels. A capsule and a thick peptidoglycan layer protect its membrane from MAC (reviewed in [1,8]). By inhibiting the central hub of the complement cascade, the C3 convertase, *S. aureus* reduces the production of C3b, C3a and C5a, interfering with both opsonization and inflammation [1,9]. These findings are in line with the previously observed role of C3 in controlling *S. aureus* bacteremia [10]. In addition, in a mouse model of *S. aureus*-septic arthritis, deficiency of C3 diminishes opsonization and phagocytosis of *S. aureus* and thereby impairs host defense [11].

Considering adaptive immunity, there is compelling evidence that antibodies contribute to clinical protection from *S. aureus* infection. Most human adults have a broad spectrum of *S. aureus-* specific antibodies in their body fluids with large inter-individual variation in terms of antibody titers and the spectrum of *S. aureus* antigens that are recognized [12,13]. High titers of specific antibodies are associated with a reduced risk of *S. aureus* infection and/or a less severe disease course [14]. Conversely, hyperimmunoglobulin E syndrome (HIES) patients are highly susceptible to recurrent *S. aureus* infection. In the majority of cases the disease is caused by heterozygous missense mutations and short deletions in signal transducer and activator of transcription 3 (*STAT3*) leading to an impairment of T cell development, in particular of Th17 cells. Recently it was shown that HIES patients also have very low anti-*S. aureus* antibody titers, although total serum IgG levels are in the normal range. Presumably, this is due to their impaired T cell response, which we discuss in detail below. Immunoglobulin (Ig)G replacement therapy significantly ameliorates *S. aureus* control, with concomitant antibiotic treatment, which makes a strong case of a protective role of antibodies [15,16].

Binding of IgG antibody to Fc receptor on phagocytes can opsonize the bacteria, whereas binding of IgG and IgM to bacteria triggers the complement cascade. Moreover, antibodies can neutralize *S. aureus* toxins and other virulence factors [12].

Recent studies have highlighted the importance of T cell-mediated immune response in *S. aureus* clearance. In a mouse model of persistent *S. aureus* infection, deficiency of T cells increased the susceptibility to *S. aureus* [17]. In addition, in murine models of nasal colonization and cutaneous infection, production of interleukin (IL)-17A by Th17 cells is required for bacterial clearance by promoting neutrophil influx to the site of pathogen invasion [18]. Furthermore, as discussed before, HIES patients with a defect in the STAT3 signaling pathway, display impaired Th17 differentiation and are highly susceptible to recurrent severe infections with *S. aureus* [19].

It is well known that most B cells require help by T cells to generate high affinity antibodies, such that the observation of a broad *S. aureus*-specific antibody repertoire indicates the existence of numerous *S. aureus*-specific T cells. Several recent studies have provided evidence for robust CD4+ and CD8+ T cell memory of staphylococcal antigens in humans [20,21,22].

There is limited knowledge about the mechanisms by which *S. aureus* activates the T cells and directs their differentiation into effector and memory T cell subpopulations, but DCs are bound to be critically involved. DCs have a central role as antigen-presenting cells (APCs) for T cells, and they have a decisive influence on the quality of the adaptive immune reaction.

**Figure 1:**
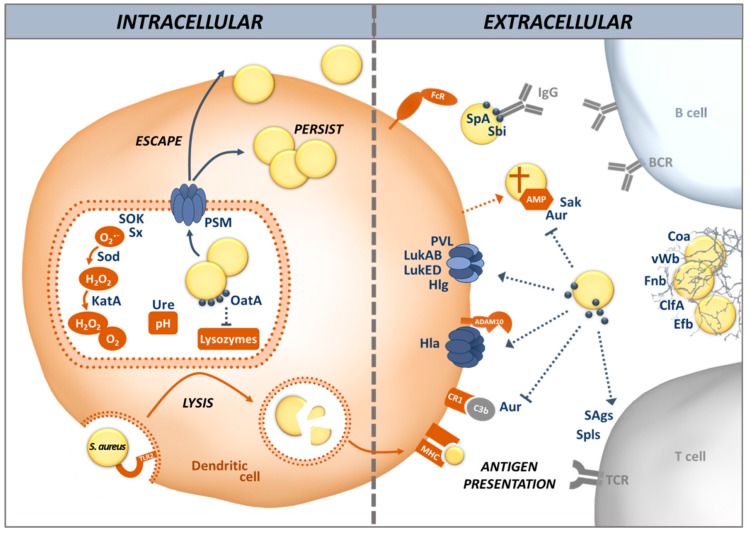
*S. aureus*’ interaction with a dendritic cell during an infection. As antigen-presenting cells DCs are able to take up *S. aureus*, lyse the bacteria and present bacterial peptides on MHC class II to initiate a specific T cell response. However, *S. aureus* displays a broad range of mechanisms to avoid opsonization, phagocytosis and proteolytic degradation by DCs. In the extracellular space, *S. aureus* avoids opsonization by blocking antibody and complement function. SpA captures antibodies via their Fc region, thereby preventing recognition by Fc receptors. Aur cleaves complement factor C3 into non-functional C3b. ClfA, Efb and FnbAB bind to fibrinogen and fibronectin, respectively, facilitating the formation of a mesh that protects *S. aureus* from phagocytosis. The coagulases Coa and vWb potentiate this process by mediating the conversion of fibrinogen into fibrin. After engulfment by phagosomes, *S. aureus* can increase the local pH by producing Ure, preventing efficient lysis. OatA acetylates the peptidoglycan cell wall, rendering *S. aureus* resistant to lysozymes. The Sx and SOK have antioxidant properties, protecting *S. aureus* from membrane damage. In addition, *S. aureus* SodA, SodM and KatA can act in cascade to detoxify ROS. PSMs enable *S. aureus* to escape from the phagosomes, thus invading the cytoplasm and possibly killing the host cell, which releases *S. aureus* into the extracellular space. AMPs secreted by DCs can be degraded by Aur and Sak, protecting *S. aureus* from being killed. Furthermore, *S. aureus* produces several pore-forming toxins, among them Hla and the bi-component toxins LukAB, LukED, PVL and Hlg, that can directly kill DCs. Finally, *S. aureus* SAgs and Spls are able to modulate the balance of the initiated T cell response towards a more favorable Th2 profile. Brown: DC factors; blue: *S. aureus* factors. Abbreviations: ADAM10: A disintegrin and metalloproteinase domain-containing protein 10; AMP: Anti-microbial peptides; Aur: Aureolysin; BCR: B cell receptor; DCs: Dendritic cells; C3: Complement factor 3; Clf: Clumping factor; Coa: Coagulase; CR1: Complement receptor 1; Efb: Extracellular fibrinogen binding protein; Hla: Alpha-hemolysin; Hlg: Gamma-hemolysin; Fc: Fragment crystallizable; Fnb: Fibrinonectin-binding protein; Kat: Catalase; Luk: Leukocidin; MHC: Major histocompatibility complex; OatA: *O*-acetyltransferase A; PSM: Phenol-soluble modulin; PVL: Panton-Valentine leucocidin (PVL); ROS: Reactive oxygen species; SAgs: Superantigens; Sod: Superoxide dismutase; SOK: Surface factor promoting resistance to oxidative killing; SpA: *S. aureus* protein A; Spls: Serine protease-like proteins; Sx: Staphyloxanthin; TCR: T cell receptor; TLR2: Toll-like receptor 2; vWb: von Willebrand factor-binding protein; Ure: Urease.

In this article, we review different aspects of DC physiology and how these cells interact with *S. aureus* during colonization and infection. We also address the countermeasures *S. aureus* uses to divert and disturb the immune response triggered by recognition of the bacteria by DCs, including the induction of allergic inflammation (illustrated in Figure 1). We place emphasis on the airway environment, because multifaceted interactions between *S. aureus* and its host take place at this site: colonization, infection and allergy.

## 2. Dendritic Cells

DCs were first described by Paul Langerhans in 1868 [23] as “branched skin cells resembling neurons”, hence the term of Langerhans cells for skin DCs. Steinmann and Cohn proposed the term “dendritic cells” in 1973 and characterized their “tree-like” morphology (Greek, *dendron*) and tissue distribution in mice [24].

DCs are highly specialized phagocytic cells. Their main function is antigen presentation to T cells, and they have the unique ability to initiate and regulate both innate and adaptive immune responses against various antigens [25]. DCs originate in the bone marrow and travel through the blood into the tissues throughout the body, including the skin, mucosal tissues and lymphoid organs [26,27,28]. As sentinel cells in the tissues, DCs continuously take up antigen, sampling their microenvironment. Upon recognition of pathogen-associated and danger-associated signals, they initiate a response of the adaptive immune system. The DCs stop taking up additional antigen and migrate from the local tissue to secondary lymphoid organs, where they differentiate into mature DCs. The latter express high levels of major histocompatibility complex (MHC) class I and II, adhesins and costimulatory molecules, and can thus act as professional APCs for T cells [29]. They efficiently activate naïve antigen-specific T cells and strongly influence their differentiation into different subsets such as Th1, Th2, Th17 or Tregs. 

In addition to their crucial role at the interface between innate and adaptive immune system as professional APCs, DCs contribute to the clearance of the opportunistic pathogen *S. aureus*. Although their ability to directly kill *S. aureus* is limited, they play a major role in the initiation and regulation of the immune response at the infection site [4,5,26,30]. By producing cytokines, DCs are essential for the recruitment of other effector cells specialized in killing bacteria, e.g., neutrophils [5,31]. In addition, IL-27 from APCs reduces the pH of phagolysosomes which boosts the ability of DCs to kill intracellular bacteria [32]. Moreover, activated platelets can stimulate DCs via CD40L production and improve their maturation, increasing cytokine secretion as well as antigen presentation in case of *S. aureus* infection [33].

DCs are generated during hematopoiesis from precursors of lymphoid or myeloid origin, which is an antigen-independent process. The progenitors in the bone marrow, called macrophage- and DC precursors (MDP), give rise to DCs and Mφs [34]. In recent years, awareness of the existence of several types of DCs has grown. Depending on their phenotype, function and tissue distribution [35,36], they are broadly classified into two major groups: conventional or classical DCs (cDCs) [24,37,38] and non-conventional DCs [39], with each group comprising more than one distinct subpopulation.

cDCs originate from pre-cDCs under the influence of granulocyte-macrophage colony stimulating factor (GM-CSF) and IL-4. This type of bone marrow-derived DCs has been extensively studied in in vitro experiments, and they are the most valuable source of knowledge about human and murine DCs. In vivo, the precursors of DCs exit the bone marrow and migrate through the blood into lymphoid tissues, bone marrow, spleen and lymph nodes, where they differentiate into cDCs characterized by the integrin CD11c [40]. Since Mφs also express CD11c, additional markers are required. Tyrosine kinase receptor “fms-like tyrosine kinase 3” (Flt3 or CD135) is an excellent marker that distinguishes cDCs from Mφs. cDCs are further divided into subgroups based on their tissue localization and cell surface markers. In mice, lymphoid cDCs cells express CD8α and CD4 (referred to here as cDC1), which constitute about 20–40% of total spleen and LN cDCs, while human cDCs cells express CD370 (Clec9A) and the chemokine receptor XCR1 [41,42]. cDCs in non-lymphoid tissues are either CD103+ CD11b− or CD103− CD11b+ (referred to here as cDC2) and lack the marker CD8. However, both CD8+ cDCs and CD11b+ cDCs proliferate in response to Flt3 (reviewed in [42]). Human and murine cDC1 cells that reside in the barrier organs such as the skin, skin-draining lymph nodes and murine Peyer’s patches, express the integrin CD103 [43].

cDCs are key players in the polarization of the T cell response. cDCs1 secrete Th1 polarizing cytokine IL-12p70, but are also capable of producing immune regulatory cytokines such as transforming growth factor beta (TGF-β) and IL-10, both important in immune tolerance [44]. cDC2 induce the Th2 immune response to helminth infections, whereas fungal pathogens induce the Th17 immune response. In humans, cDC2 cells are characterized by expression of CD103, FcR1A and the alpha-chain of the high affinity receptor for IgE [45]. Compared to cDC1, cDC2 in mouse and human are less capable of presenting antigens via MHC class II [46].

Plasmacytoid DCs (pDCs) were first reported in 1958 by Lennert et al., and named by their appearance, which resemble plasma cells [39]. Precursors of pDCs express low levels of the GM-CSF but high amounts of the IL-3 receptor, and they differentiate into pDCs in response to IL-3 [47,48]. Human pDCs are characterized by their intermediate expression of CD11c, low levels of MHC class II and high density of CD123, but lack of CD11b [49,50]. In mice, pDCs exhibit surface markers that are shared with other cell types, e.g., B220 and Ly6C [51,52]. pDCs are found circulating in the blood and in peripheral organs such as bone marrow, spleen, thymus, lymph nodes, and the liver. They are known to play an important role in the production of type I interferons (IFN)-α/β by virtue of their capacity to sense viral nucleic acids [53,54].

## 3. Recognition and Uptake of *S. aureus* by DCs

DCs can efficiently recognize a wide range of invading microorganisms. The pattern recognition receptors (PRRs), which are expressed on their surface, can sense pathogen-associated molecular patterns (PAMPs) or damage associated molecular patterns (DAMPs). Among all PRRs, the Toll-like receptors (TLRs) are particularly well studied and characterized. They play a key role in inducing both direct and indirect DC maturation. To date, 10 human TLRs (TLR1–10) and 12 murine TLRs (TLR1–9, TLR11–13) have been identified. TLR1, 2, 4–6 and 11 are expressed at the cell surface while TLR3 and 7–9 are located intracellularly in the endosomal compartments [55].

TLR2 is the main receptor involved in *S. aureus* recognition, through the detection of lipoproteins, wall teichoic and lipoteichoic acid, as well as peptidoglycan [56,57]. In association with TLR1 or TLR6, TLR2 is able to sense diacyl or triacyl lipopeptides or lipoproteins [58]. Counteracting this, staphylococcal superantigen-like proteins (SSL) 3 and 4 inhibit TLR2 through interfering with both lipopeptide binding and TLR dimerization [59]. In a murine model of infection, *S. aureus* has been shown to evade TLR2 activation by secreting SSL3, indicating that TLR2 inhibition is important for staphylococcal pathogenesis [60]. The production of phenol soluble modulins (PSMs) increases the release of lipoproteins from the surface of *S. aureus*, whereas *S. aureus* strains producing low amounts of PSMs are less detected by TLR2 [56]. In AD, a chronic allergic inflammatory disease of the skin, DCs are less responsive to TLR2 stimulation, which could blunt their anti-*S. aureus* activity [61]. Colonization of lesional skin by *S. aureus* occurs in 90% of AD patients aggravating the inflammation and sometimes leading to severe invasive infections such as endocarditis or bacteremia [62]. TLR8 in MOs, Mφs and DCs senses *S. aureus* RNA, a vita-PAMP enabling the assessment of microbial viability [63,64]. TLR9 binds *S. aureus* CpG-DNA and induces type I IFN signaling [65]. In osteoblasts, TLR9 also improves the killing of *S. aureus* by increasing the production of reactive oxygen species (ROS) [66]. Persistent *S. aureus* carriers have a higher expression of TLR9 that is dependent on the carrier’s TLR9 haplotype, sex and hormone status, which could explain why women are more susceptible to *S. aureus* septicemia than men [67,68].

To escape PRR-triggered phagocytosis and avoid the reaction of host cells to danger signals, *S. aureus* produces many proteins enabling it to invade different cell types, including non-phagocytic cells, via a zipper-type mechanism [69,70]. This is mediated by adhesins, known as microbial surface components, which recognize adhesive matrix molecules (MSCRAMMs) [71]. MSCRAMMs, including the fibronectin binding proteins (Fnb) A and B, are covalently linked to the peptidoglycan cell wall and involved in adhesion of *S. aureus* to the host cell matrix (Figure 1). *S. aureus* Fnb A and B bind to the host cell integrin α_5_β_1_ via a fibronectin bridge. Other MSCRAMMs such as clumping factor (Clf) A and B are also involved in the attachment of *S. aureus* to host cells. Protein A (SpA), in contrast, interacts directly with host cell receptors [72]. These mechanisms stimulate actin rearrangement in the host cell and *S. aureus* is internalized without triggering TLRs [70,73,74].

Other adhesins, known as secretable expanded repertoire adhesive molecule (SERAMs), are released by *S. aureus* and re-attach to the bacterial surface non-covalently. The autolysin (Alt) family of proteins, AltA and Aaa, mediate adherence of *S. aureus* to host components such as fibronectin, gelatin and heparin, facilitating colonization and infection [75]. Among the SERAMs, the extracellular adherence protein (Eap) and extracellular matrix and plasma binding protein (Emp) have been shown to bind to host extracellular matrix components and plasma proteins, such as fibrinogen, fibronection, vitronectin, collagen, as well as to the endothelial cell adhesion receptor ICAM-1 [76,77]. Other SERAMs exploit the host’s coagulation system. Staphylocoagulase (Coa) and von Willebrand factor-binding protein (vWbp) activate prothrombin by inducing a conformational change to form a complex that cleaves fibrinogen into fibrin. ClfA then binds to the resulting fibrin cables to form a mesh, thus protecting *S. aureus* from phagocytosis and inducing abscesses. In this way, *S. aureus* can aggregate and shield themselves from phagocytes in a tight network thanks to bacterial and host proteins [78].

Opsonization is an efficient way to help phagocytes, including DCs, to take up bacteria and target them for killing. Igs and complement components are the main opsonins in the body fluids. Phagocytes possess specific receptors for IgG as well as the main complement opsonin C3b. DCs express Fcγ receptors on their surface that bind to IgG, helping the DC to immobilize their target for phagocytosis [79]. However, *S. aureus* possesses a broad range of factors that can prevent opsonization, either by hiding the targets of the opsonins or by using decoys. Many *S. aureus* strains are encapsulated by sugar polymers that cover most of the immunogenic surface-exposed proteins [80]. The two main serotypes are capsular polysaccharide (CP) 5 and CP8, which represent about 75% of all clinical isolates. Although strains producing a capsule are more resistant to phagocytosis, this resistance is overcome when specific Igs bind to the capsule polymers [81]. Interestingly USA300, one of the main CA-MRSA clones, does not display any capsule, demonstrating that capsule targeting vaccines cannot cover the whole diversity of *S. aureus* clones. Moreover, all *S. aureus* clinical isolates produce SpA, a major virulence factor, which binds with high affinity to the Fc-portion of Igs, mostly IgG, rendering them unable to bind to the bacterial surface in the correct orientation and thus preventing opsonization [82]. SpA can also act as a B cell superantigen, since it binds to B cell receptors which use the V_H_III element, inducing apoptotic cell death. This can result in the complete deletion of the respective B cell clones [83].

DCs possess CR3 (CD11b/CD18) and CR4 (CD11c/CD18), such that coating of *S. aureus* with C3b facilitates phagocytosis [84]. However, *S. aureus* is adept in complement evasion. Many secreted proteins such as SpA, aureolysin (Aur), staphylokinase (Sak), extracellular fibrinogen-binding protein (Efb), and staphylococcal complement inhibitor (SCIN) interact with components of the complement pathways, preventing this system from fulfilling its purpose in opsonization and killing of pathogens (Reviewed in [1,85]). For instance, Aur cleaves C3 to produce a non-functional C3b fragment, hence preventing a normal activation of the complement and its opsonizing effects [86]. Furthermore, Sak and Aur can also bind and degrade secreted antimicrobial peptides such as α-defensins and LL-37, before these can cause pores in the bacterial membrane [87,88,89].

To kill before being killed is another strategy of *S. aureus*, which is effective even before the bacteria are taken up by the host cells. *S. aureus* produces bi-component pore-forming toxins called leukocidins (Luks) which play a pivotal role in killing host immune cells, including DCs. The target structures of these toxins on host cells have been identified over the past few years (reviewed in [90]). LukED targets the C-C chemokine receptor (CCR) 5 as well as the C-X-C motif chemokine receptor (CXCR) 1 and CXCR2, to kill DCs, T cells and Mφs [91,92]. LukAB specifically binds to the CD11b I domain in human but not murine polymorphonuclear cells (PMNs) and allows *S. aureus* to either kill its host cell or escape from phagosomes [93]. Since some subpopulations of DCs express CD11b, as part of the CR3, they can be targeted by LukAB [42,93,94,95]. LukMF’ is mostly found in *S. aureus* isolates from ruminants and is associated with bovine infections. It targets CCR2 and CCR5 on bovine and CCR1 on both human and bovine neutrophils and induces cell death [96]. These CCRs are also found on human DCs, which make them vulnerable targets [97]. The specificity of gamma-hemolysin (Hlg) depends on the subunits forming the toxin. The heterodimer HlgAB has a hemolytic function by binding to the Duffy Antigen Receptor for Chemokine (DARC) on erythrocytes and can also target CXCR1, CXCR2 and CCR2 on DCs [97,98,99]. HlgCB binds to the same targets as Panton-Valentine leukocidin (LukSF-PV, also known as PVL), C5aR and C5L2 [98]. Moreover, some toxin subunits can cross-interact with others and form hybrid toxins, which might increase the number of targets on the surface of host cells. For instance, HlgB can compete with LukS-PV and LukD to interact with LukF-PV [100,101].

## 4. *S. aureus* Evades Killing by DCs as well as Antigen Processing and Presentation 

After internalization by phagocytosis, *S. aureus* is exposed to bactericidal effector mechanisms in DCs. Phagosomes mature and fuse with lysosomes containing hydrolases. In the phagolysosomes, *S. aureus* is also subjected to oxidative conditions rich in ROS and reactive nitrogen species (RNS). NADPH oxidase (NOX2) consumes oxygen to produce superoxide radical anions (O_2_^•−^) and hydrogen peroxide (H_2_O_2_). Oxidation modulates the activities of different groups of proteases and thus reduces proteolysis within phagosomes of DCs [102]. In addition to this oxidative stress, *S. aureus* is also submitted to acidic pH. Acidification of phagosomes in DCs is reduced compared to Mφs or neutrophils because the responsible enzyme, the V-ATPase, is incompletely assembled in immature DCs [30]. Proteases are therefore less active such that proteins are only partially degraded [103]. While reducing the ability of DC to truly clear the bacteria, incomplete protein degradation permits DCs to expose a higher diversity of peptides on MHC class II to efficiently prime T cell responses. Depending on the signals from their microenvironment, most importantly the nature of the infectious agent, DCs release various cytokines that direct the differentiation of naive CD4+ T cells into different effector and memory T cell subsets.

However, in many cases *S. aureus* manages to survive within eukaryotic host cells, including professional and non-professional phagocytes [104]. Acidification of the phagolysosome is counteracted by secretion of urease, which increases the pH by hydrolyzing urea into ammonia [105]. *S. aureus* is also highly resistant to oxidative stress, because staphyloxanthin (Sx), the main pigment of *S. aureus*, works as an antioxidant and prevents membrane peroxidation [106]. SOK (surface factor promoting resistance to oxidative killing) displays similar properties and is considered a virulence factor in endocarditis [107]. SodA, SodM and KatA act in cascade to detoxify O_2_^-^ into H_2_O_2_ and then into H_2_O + O_2_ [108,109]. A recently discovered molecule, the staphylococcal peroxidase inhibitor (SPIN), inhibits myeloperoxidase (MPO) in neutrophils and protects *S. aureus* from oxidative stress during phagocytosis [110]. DCs lack MPO, but they are influenced by the neutrophil-derived enzyme, which inhibits antigen uptake and processing by DCs, as well as their migration to lymph nodes and, as a consequence, T cell priming [111,112]. MPO-inhibition by SPIN could, therefore, enhance *S. aureus* survival in neutrophils with a trade-off: promoting the induction of an adaptive immune response by DCs.

Modification of the bacterial cell wall is another way to avoid degradation. The *O*-acetyltransferase A (OatA) adds an acetyl group to *N*-acetylmuramic acid in the peptidoglycan, rendering *S. aureus* resistant to the lysozyme produced in the phagosome [113]. This may be one reason why lipoproteins that are embedded in the cell wall do not elicit a strong adaptive immune response [114].

Moreover, *S. aureus* has means to destroy the phago(lyso)some membrane and escape into the cytoplasm. Upon internalization by professional phagocytes, the bacteria produce phenol-soluble modulins (PSMs), similar to the delta hemolysin, which can form membrane pores. In DCs, PSMs help *S. aureus* to escape from the phagosome, invade the cytoplasm and possibly kill the host cell [115,116,117]. PSMs are under the positive control of the *agr* system, a global regulator that is active in the phagolysosome environment [69]. This mechanism could allow *S. aureus* to interfere with antigen processing and presentation on the MHC class II of the DCs, reducing their T cell-priming ability. Another effect of PSMs is the modulation of cytokine production by the host cells [118,119,120]. In DCs, the activation of the p38-CREB pathway by the PSMs induces a tolerogenic phenotype with a reduction of TLR2 signaling and production of inflammatory cytokines, leading to an increased priming of anti-inflammatory T_regs_ [118,119]. After escaping the phagosome, *S. aureus* is able to replicate in the cytosol and induce host cell death or survive in a dormant state for extended time periods in the shape of small colony variants (SCV) [69]. This ability is probably linked to the chronicity of infections. It is still unclear what determines the balance between killing the host cell and surviving inside. Chronic *S. aureus* infection are very difficult to treat, and novel approaches are required. Chloroquine improved clearance of *S. aureus* from lung epithelial cells in combination with antibiotic therapy [121,122].

Autophagy or, more precisely, xenophagy, is used by DCs to kill pathogens and present antigens, once the bacteria have escaped into the cytoplasm. The cytoplasmic invaders become surrounded by double-membrane vacuoles, named autophagosomes, which present microtubule-associated protein 1 light chain 3 (LC3) associated to phosphatidylethanolamine (LC3-II). These fuse with lysosomes, redirecting their cargo from the cytoplasm back into the lysosomal pathway, followed by digestion and presentation of antigenic proteins. Consequently, peptides derived from cytoplasmic antigens, which are usually presented on the MHC class I, are loaded onto the MHC class II molecules, a process known as cross-presentation of antigens.

The *agr* system conditions *S. aureus* survival during autophagy by preventing the fusion between autophagosomes and lysosomes. *S. aureus* strains with a highly active *agr* system are not killed in autophagosomes in vitro and display an extended intracellular survival within phagocytes in vivo [31]. *S. aureus* can prevent fusion of autophagosomes with lysosomes via a novel mechanism, involving activation of MAPK14 and ATG5 phosphorylation [123]. Autophagy can tolerize host cells to the effects of alpha-toxin (alpha-hemolysin, Hla), another pore-forming toxin produced by *S. aureus* under *agr* control [124]. Indeed, autophagy allows cells to recycle membranes affected by Hla and endure higher concentrations of pore-forming toxins. Remarkably, Hla has been identified as an important autophagy-inducing factor [31,125]. 

As previously mentioned, DCs produce cytokines to control the immune response, recruit immune cells on the infection site and activate T cells. *S. aureus* is able to modulate the cytokine production of immune cells with several mechanisms. The ESAT-6-like secretion system (Ess) is encoded in the conserved *ess* gene cluster [126]. The *ess*-encoded virulence factor EsxA dampens the pro-apoptotic response in infected DCs and may allow *S. aureus* to use these cells as a Trojan horse. EsxB dampens the production of regulatory and pro-inflammatory cytokines by the infected DCs, resulting in a reduced production of IFN-γ and IL-17 by activated CD4+ T cells [127].

*S. aureus* can also induce an overstimulation of the immune system by secreting superantigens [128]. The species harbors 25 genes encoding superantigens, comprising the toxic shock syndrome toxin-1 (TSST-1) and the staphylococcal enterotoxins, organized in the “enterotoxin gene cluster” *egc* or on mobile genetic elements [129,130]. As superantigens, they are able to interact with the MHC class II on the DCs and the Vβ region of the T cell receptor (TCR), bypassing the conventional antigen specific activation of T cells, to activate up to 20% of the T cell population [131]. 

## 5. Interaction of DCs with *S. aureus* in the Respiratory Tract

The airways are a site of extensive interaction between *S. aureus* and its host with extremely diverse outcome. Around 25 to 35% of adults persistently carry *S. aureus* in the nose, while the remainder is able to rapidly clear the bacteria from the upper airways [132]. However, *S. aureus* can also cause pneumonia, a life-threatening infection of the lungs. It may be community acquired, often in the context of an influenza infection, in which case the mortality rate can increase to 50% [133]. In hospitals, patients receiving mechanical ventilation are vulnerable to *S. aureus* infection and develop so-called ventilation-associated pneumonia. There is increasing evidence that, besides commensal and invasive behavior, *S. aureus* may also drive allergic airway inflammation [134,135]. Colonization with *S. aureus* is associated with childhood wheezing and asthma [136]. van Zele et al. have found that 66.7% of patients with nasal polys and co-morbid asthma are colonized with *S. aureus*, in contrast to 33.3% of healthy adults. In case of additional aspirin hypersensitivity, these were even 87.5% [137]. Moreover, IgE antibodies specific for *S. aureus* enterotoxins and serine protease-like proteins were found in asthmatic patients [138,139].

DCs play a critical role in shaping the adaptive immune response at mucosal sites. The modulation of the T helper cell response to *S. aureus* infection by lung DCs is of particular interest. Over the past years, many subsets of DCs have been described in lung immunity. Under steady state conditions, “immature” DCs in the lungs efficiently recognize and capture inhaled materials. DCs that have encountered antigens or allergens undergo maturation, leave the lung and migrate to draining regional lymphoid tissues, where they present the processed antigenic peptides to naïve T cells. This results in T cell activation and polarization, depending on the nature of the antigen [140]. It has been well documented that DCs induce protective immune responses against pathogens, but may also initiate inflammatory immune responses to harmless allergens, being thus involved in the pathophysiology of asthma and allergic rhinitis [141]. Both protection and allergy are relevant in the interaction of *S. aureus* with its host.

*S. aureus*-primed cDCs are highly responsive and induce T cell differentiation into IFN-γ-producing CD4+ (Th1) and CD8+ (Tc1) cells [142]. In fact, healthy donors and patients show a large pool of *S. aureus*-specific memory T cells that respond to *S. aureus* with the secretion of IFN-γ and/or IL-17 [21,22]. The existence of CD8+ T cell memory cells and their responses against staphylococcal antigens are important for minimizing inflammation and promoting T cell tolerance [20]. Moreover, long-term exposure of mice to *S. aureus* failed to produce IL-2 after an antigen-specific T cell response, suggesting that T cells undergo anergy during persistent infection [143]. The lungs are vulnerable to inflammation-induced organ damage interfering with gas exchange, which may rapidly become critical. A strong T cell response driven by DC recognition of *S. aureus* is therefore a double-edged sword, as has been demonstrated in murine pneumonia models (reviewed in [144]).

*S. aureus* colonization of the airways is associated with allergic airway disease, but the mechanisms of allergic sensitization or exacerbation by *S. aureus* are still poorly understood [136,145]. Asthma is defined by chronic airway inflammation with reversible airway obstruction, airway hyperresponsiveness, infiltration Th2 cells and eosinophils into the airway submucosa, mucus hypersecretion and airway remodeling. It has been more than two decades since Robinson et al. and other groups demonstrated that atopic asthma was associated with activation of Th2 type of T cell in the airways [146]. In addition, in murine model of asthma and allergy, adoptive transfer of Th2 cells, but not Th1 cells, induces airway hyper-responsiveness (AHR) [147]. The involvement of DCs in asthma was characterized in 1998 by Lambrecht et al. [148], who showed that cDCs were essential for triggering allergy in ovalbumin-sensitized mice (OVA). Conversely, pDCs were protective [141]. Recently, it has been demonstrated that a subtype of cDCs, CD117+ CD172α+, is a major mediator of inflammation in asthma by promoting the induction of Th2 immunity in spleens [149].

Some airborne allergens such as Der p1 from house dust mites disrupt the epithelial barrier by cleaving the tight junction proteins, thus gaining access to the DCs at the basolateral side of the epithelium [150]. It is tempting to speculate that *S. aureus* alpha-toxin (hemolysin alpha, Hla) may act similarly, because the pore-forming toxin activates the host metalloprotease ADAM10, which destroys epithelial adherens junctions [151,152]. Moreover, ADAM10 and its ligand Notch1 were shown to be essential for DCs to produce Th2 type cytokines in a murine model of IgE-mediated anaphylaxis, suggesting that ADAM10 activation by Hla could have pro-allergenic effects [153]. On the other hand, human Hla-specific T cells release mainly IFN-γ and IL-17, indicating that the toxin itself is not an allergen [21,154,155,156]. It is not known how Hla affects DCs, but in monocytes the toxin is able to induce IL-17, which is in line with the observed cytokine profiles of Hla-specific T cells [151]. While questions remain regarding the possible pro-allergenic effects of Hla, there can be no doubt that the toxin is a decisive virulence factor in *S. aureus* pneumonia [151,157]. Via ADAM10 the toxin induces the secretion of pro-inflammatory cytokines and cell death via the nucleotide-binding domain and leucine-rich repeat containing gene family, pyrin domain containing 3 (NLRP3) inflammasome [158]. In the lungs, epithelial tissue destruction could provide host-borne nutrients for bacterial growth, which may be one reason for the disastrous effects of Hla in pneumonia [159]. Neutralization of *S. aureus* alpha toxin is under development as adjunct therapy with standard antibiotic treatment [151,160].

It was discovered that two classes of *S. aureus* proteins, SEs and serine protease-like proteins (Spls), readily cause allergic sensitization in the airways [161]. Affected humans and experimental animals elaborate antigen-specific IgE as well as Th2 cells [139,162]. This demonstrates that different *S. aureus* factors can elicit adaptive immune responses of different quality in the same individual. Similar observations have recently been reported for fungal antigens by Bacher et al. [163]. We conclude that many microbial proteins have adjuvant activity, determining the cytokine and antibody profiles of the specific T and B cells. As this is an emerging topic, there are many open questions: How are DCs involved in the process? Are the superantigenic or enzymatic activities of the staphylococcal factors important for their allergenic properties? Are there general features of bacterial allergens? How does intrinsic adjuvanticity of bacterial antigens affect the outcome of vaccination, especially, if non-adjuvanted bacterial factors are used as vaccines? Is *S. aureus* able to initiate the allergic march in susceptible individuals, or does it merely exacerbate pre-existing allergic inflammation? We hypothesize that *S. aureus* allergens may sensitize vulnerable persons whose allergic reaction is then potentiated by bacterial toxins and the PAMPs in the airways.

## 6. Conclusions

Coordination of the immune response at the interface between innate and adaptive defense mechanisms is an essential function of DCs. These phagocytic leukocytes sense microorganisms in tissues which border on the external environment. Not surprisingly, *S. aureus* has evolved means to prevent phagocytosis, to resist killing inside phagosomes, and manipulate DCs to its advantage. The multifaceted interactions between *S. aureus* and its host take place in the airways. The outcomes range from rapid clearance through symptom-free colonization to asthma or life-threatening pneumonia. Remarkably, single *S. aureus* proteins can elicit immune responses of distinctive cytokine and antibody profiles in the same individual, demonstrating that bacterial antigens have adjuvant properties. It will be worthwhile exploring more closely how DCs affect these processes and how their response is determined by various *S. aureus* virulence factors. A better understanding of the behavior of DCs, pivots of the immune system exhibiting great plasticity, will also benefit vaccine research.
